# Associations Between Stress-Coping Strategies and Treatment Discontinuation in Mood Disorders

**DOI:** 10.1192/j.eurpsy.2026.10596

**Published:** 2026-07-31

**Authors:** E. Moon

**Affiliations:** Psychiatry, Pusan National University School of Medicine, Busan, Korea, Republic Of

## Abstract

**Introduction:**

Long-term treatment is crucial for achieving favorable outcomes in mood disorders. However, the prolonged treatment process can be stressful, and the ways in which patients cope with stress may influence whether they adhere to or discontinue treatment.

**Objectives:**

This study aimed to investigate the associations between stress-coping strategies and treatment discontinuation in patients with mood disorders.

**Methods:**

A total of 321 patients diagnosed with mood disorders were included. Dropout rates were estimated at 1, 2, and 3 years. The associations between stress-coping strategies and treatment discontinuation were analyzed using a Cox regression model.

**Results:**

The cumulative discontinuation rates were 31.2%, 40.5%, and 47.0% at 1, 2, and 3 years, respectively. Younger age (p = 0.043), emotional coping (p = 0.020), and compulsive coping behaviors (p = 0.043) were significantly associated with treatment discontinuation.

**Conclusions:**

Despite the clinical importance of sustained treatment in mood disorders, nearly half of patients discontinued care within three years. The findings suggest that specific stress-coping strategies may contribute to treatment discontinuation, and that addressing maladaptive coping behaviors could improve adherence and long-term treatment outcomes.

**Disclosure of Interest:**

None Declared

